# Engineering Microbes to Bio-Upcycle Polyethylene Terephthalate

**DOI:** 10.3389/fbioe.2021.656465

**Published:** 2021-05-28

**Authors:** Lakshika Dissanayake, Lahiru N. Jayakody

**Affiliations:** ^1^School of Biological Sciences, Southern Illinois University, Carbondale, IL, United States; ^2^Fermentation Science Institute, Southern Illinois University, Carbondale, IL, United States

**Keywords:** synthetic microbes, polyethylene terephthalate, PET degradation, metabolic engineering, bio-upcycling

## Abstract

Polyethylene terephthalate (PET) is globally the largest produced aromatic polyester with an annual production exceeding 50 million metric tons. PET can be mechanically and chemically recycled; however, the extra costs in chemical recycling are not justified when converting PET back to the original polymer, which leads to less than 30% of PET produced annually to be recycled. Hence, waste PET massively contributes to plastic pollution and damaging the terrestrial and aquatic ecosystems. The global energy and environmental concerns with PET highlight a clear need for technologies in PET “upcycling,” the creation of higher-value products from reclaimed PET. Several microbes that degrade PET and corresponding PET hydrolase enzymes have been successfully identified. The characterization and engineering of these enzymes to selectively depolymerize PET into original monomers such as terephthalic acid and ethylene glycol have been successful. Synthetic microbiology and metabolic engineering approaches enable the development of efficient microbial cell factories to convert PET-derived monomers into value-added products. In this mini-review, we present the recent progress of engineering microbes to produce higher-value chemical building blocks from waste PET using a wholly biological and a hybrid chemocatalytic–biological strategy. We also highlight the potent metabolic pathways to bio-upcycle PET into high-value biotransformed molecules. The new synthetic microbes will help establish the circular materials economy, alleviate the adverse energy and environmental impacts of PET, and provide market incentives for PET reclamation.

## Introduction

Plastic, a synthetic polymer, plays a vital role in modern life due to its versatility, advantageous material properties, and low production cost. It has been estimated that about 5–13 million tons of plastic is ended up in the ocean annually, and 5 trillion plastic particles are estimated to float in Earth’s oceans, which injures and kills marine life (Eriksen et al., 2014; Law et al., 2020). Although plastic is less prone to biodegradability, it can be partially fragmented to microplastic (5 mm to 1 μm) particularly by ultraviolet radiation, and microplastics have invaded not only terrestrial and marine ecosystem, but atmospheric ecosystems as well (Eriksen et al., 2014; de Sá et al., 2018; Allen et al., 2019). Microplastics enters the food chain, spreads toxins, and poses a potential threat to human health (Wang et al., 2018). The systematic presence of synthetic micro polymers is threatening to create a global-scale environmental crisis (Thompson et al., 2009; Jambeck et al., 2015; Geyer et al., 2017).

Polyethylene terephthalate (PET) is a thermoplastic polyester of terephthalic acid (TPA) and ethylene glycol (EG) monomers (Kim and Lee, 2012). The wide applicability of PET in various industries such as in packaging, textiles, electrical and electronics, and automotive industry is related to its properties such as high mechanical strength, light weight, electrical insulating properties, chemical inertness, and gas and moisture barrier properties (Webb et al., 2013). Global PET fiber and resin production was estimated to be around 77 million tons in 2015 (Fiber Economics Bureau, 2015; Plastic Insight, 2016). Mismanagement of PET waste contributes to the plastic pollution and demanding techno-economically feasible end-of-life or circular economy options for PET, including recycling post-consumer PET back to the original material (Hamid et al., 2018; Meys et al., 2020). Mechanical conversion of PET to the same use often results in PET polymer with poorer mechanical and structural properties, and thus, lower value by 33% (Awaja and Pavel, 2005). PET can be chemically recycled via full breakdown to monomers and repolymerized back to PET; however, chemical-based recycling costs of PET to remake the same polymer is not economically feasible (Rahimi and García, 2017; Vollmer et al., 2020). However, It has been predicted that recycled PET over virgin PET has remarkable energy and environmental impact on reducing greenhouse gas (GHG) emissions by 1.5 CO_2_-eq-ton/recycle PET and energy input over virgin PET by >20 MJ/ton (Rorrer et al., 2019).

The novel discovery of PET depolymerizing enzymes has transformed the field to develop a techno-economically feasible bio-based PET recycling process (Wei and Zimmermann, 2017; Tournier et al., 2020; Zimmermann, 2020). Researchers have uncovered novel PET hydrolases from the microorganism in plastic ecosystems (Plastisphere) and investigated them to establish bio-based PET recycling approaches (Mueller, 2006; Kawai et al., 2020). The CARBOIS, a green chemistry company, developed industrially applicable enzyme-based recycling technology to remake PET bottles with similar material properties only using recycled PET monomers (Tournier et al., 2020). Comprehensive review articles have been published on bio-based PET recycling techniques (Wei and Zimmermann, 2017; Blank et al., 2020; Ru et al., 2020; Wei et al., 2020; Kawai, 2021). With the advances in synthetic microbiology, the development of sustainable microbial-based “PET upcycling” toward a green route of the circular economy becomes attractive. Upcycling is achieved by adding value to the PET waste by providing a path for utilizing PET-derived compounds to manufacture high-value chemicals and materials (Kenny et al., 2008; Rorrer et al., 2019; Blank et al., 2020; Sohn et al., 2020). Microbial cell factories have been tailored to the deconstruction of PET in concert with the chemical processes (i.e., hybrid biochemical process). PET-derived monomers can be biotransformed into high-value platform chemicals and biomaterials, including bioplastic PET alternatives. It enables the creation of a circular material economy for PET (Sohn et al., 2020; Tiso et al., 2020). Hence, this mini-review highlights the current progress on microbial-based PET upcycling.

## Selective Degradation of PET by Microbial Enzymes

The conventional culture-dependent methods, cutting-edge multi-omics-based systems biology approaches, and molecular biology techniques enable researchers to identify novel PET-hydrolyzing enzymes from the plastisphere to cleave ester bonds of PET (Supplementary Table 1). Also, computational and machine learning approaches enable the researcher to discover novel potent PET enzymes from metagenomics databases (Danso et al., 2018; Furukawa et al., 2019; Subramanian et al., 2019). These enzymes belong to esterase, cutinase, and lipase families, and may evolve in a PET-rich environment (Roager and Sonnenschein, 2019; Alam et al., 2020; Maurya et al., 2020). The discovery of the bacterium *Ideonella sakaiensis* harboring hydrolyzing enzymes PETase and MHETase has revolutionized the field. These enzymes can completely degrade the synthetic polymer PET to its monomers TPA and EG at ambient temperature (Yoshida et al., 2016; Carr et al., 2020). Employing biological catalysis for commercial PET depolymerization is challenging due to the limited accessibility of polymer’s high crystalline ester linkages. Hence, researchers engineer the PET-hydrolyzing enzymes to enhance the activity by in-depth structure/activity relationships studies (Supplementary Table 2). For instance, (1) narrowing the binding cleft of PETase, an α/β-hydrolase fold enzyme via mutation of two active-site residues to conserved amino acids in cutinases (i.e., S238F/W159H), enhanced the crystalline PET degradation to convert PET to mono-(2-hydroxyethyl) terephthalate (MHET), and (2) the second enzyme MHETase, a specific lid-domain-containing esterase, hydrolyzes MHET to TPA and EG, but not bis-(2-hydroxyethyl) terephthalate (BHET) natively (Austin et al., 2018; Knott et al., 2020). Palm and coworkers successfully demonstrated structure-guided alterations of MHETase to active BHET by introducing three mutations to the lid domain of the MHETase (R411A/S419G/F424N) (Palm et al., 2019).

Researchers developed PET hydrolyzing enzymes acting near or above the glass transition temperature, Tg of PET (67–81°C) maximizes the PET polyester chain mobility and ester bond hydrolyzing reaction. To increase the thermal stability of TfCut2, binding sites of Ca^2+^ and Mg^2+^ are identified as potential targets for engineering (Then et al., 2015). Introduction of a disulfide bridge to substitute TfCut2 Ca-binding site increased thermal stability and activity against PET (Then et al., 2016). Thermostability of leaf-branch compost cutinase (LCC) is improved to 94.5°C by replacing the divalent metal site with a disulfide bridge (D238C/S283C). The loss of enzyme activity is restored by introducing additional mutation of residue in contact with the PET substrates (F243I) (Tournier et al., 2020). The engineered enzymes enable industrially relevant PET recycling to manufacture PET bottles with similar material properties using recovered TPA. Indeed, they achieved a 90% conversion of pre-treated post-consumer PET in less than 10 h, with a mean productivity of 16.7 g TPA L^–1^ h^–1^ with a yield of 27.9 g TPA g enzyme^–1^, and demonstrate the green route of the circular economy. In concert with computation studies, protein engineering shows the potential to develop efficient PET-hydrolyzing enzymes with improved crystalline PET activity, expanded substrate specificity, alleviated product inhibitions, and thermostability (Cui et al., 2021).

A dual enzyme system consisting of a polyester hydrolase, TfCut2 or LCC, and a carboxylesterase TfCa from *Thermobifida fusca* KW3 has been tested to overcome the inhibition of MHET on PET degradation (Barth et al., 2016). With the addition of TfCa, the total products reported an increase of 91% with TfCut2 and 104% increase with LCC. The results indicated the successful use of TfCa as a secondary biocatalyst to improve PET degradation by increased hydrolysis of MHET. Similarly, the synergistic effect on PETase and MHETase enhances the PET degradation activity, and a higher MHETase load further improved the degradation rate. Furthermore, MHETase and PETase’s chimeric enzymes (MHETase C terminus connected to PETase via flexible glycine-serine linkers) outperformed the degradation rate of unlinked PETase and MHETase (Knott et al., 2020). *Pichia pastoris* has shown potential as a better expression host for PET-hydrolyzing enzymes such as LCC and Thc_Cut1 (Gamerith et al., 2017; Shirke et al., 2018). The engineered thermophilic *Clostridium thermocellum* expressing the thermophilic PET hydrolase LCC enables the selective degradation of PET at 60°C and outperformed whole-cell-based PET biodegradation systems that employ mesophilic bacteria or microalgae. A recent study reported that expression of BhrPETase in *Bacillus subtilis* showed higher activity on amorphous PET compared to LCC and PETase, and this is the most thermostable PET hydrolase reported to date (Xi et al., 2021).

The selection of an appropriate host strain is necessary to obtain the active enzymes, and codon optimization of genes is vital to produce accurately folded soluble protein (Angov, 2011). It has been shown that TfCut2 expressed in *B. subtilis* is more efficient and thermostable than when expressed in *Escherichia coli*, and the enzymes can be used to depolymerize post-consumer PET food packaging effectively (Wei et al., 2019). Additionally, *B. subtilis* successfully employs LCC and IsPETase, methylotrophic yeast, *P. pastoris* for LCC, and cutinase from *Thermobifida cellulosilytica* (Gamerith et al., 2017; Shirke et al., 2018; Xi et al., 2021). Notably, for the commercial production of PET-hydrolyzing enzymes, the strains that allow secretory high-level expression should be used to avoid additional costly purification steps (Su et al., 2013). Since enzyme expression and purification add extra cost to the process, researchers developed the whole-cell microbial catalysts to degrade the PET by heterologous expression of PET-hydrolyzing enzymes (Samak et al., 2020). A promising strategy to overcome the problem of PET waste in marine environments where an engineered photosynthetic marine microalgae *Phaeodactylum tricornutum* with the ability to produce and secrete an improved PETase into the culture medium has been developed (Moog et al., 2019). Seo and coworkers successfully fused the PETase with *E. coli* SRP-dependent signal peptides to enable the secretion of PETase via a sec-dependent secretion system and demonstrated the PET degradation activity by engineered *E. coli* (Seo et al., 2019). Expressing PETase on yeast’s cell surface shows new insights into developing whole-cell eukaryotic systems for efficient degradation of highly crystalline PET (Chen et al., 2020). The cell surface display of bacterial PETase on *P. pastoris* showed a 36-fold turnover rate compared to purified PETase, showing a promising approach toward whole-cell biocatalysts for efficient biodegradation of PET. In sum, biorecycling enables the researcher to obtain the original monomers of PET to upcycle into value-added chemicals and materials via synthetic biocatalysts.

## Metabolic Routes to Upcycle PET-Derived Substrates

**FIGURE 1:**
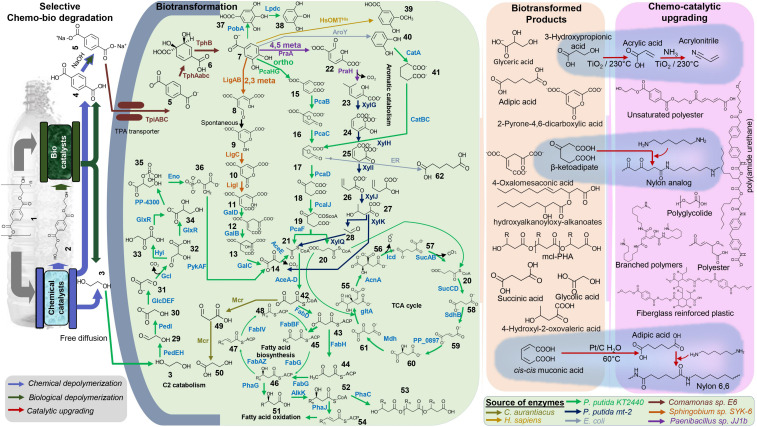
Overview of hybrid biochemical upcycling of PET. The potential bacterial biofunneling pathways of selectively degraded PET products, TPA, and EG into various economically valuable compounds and materials are highlighted. Examples of high-value biotransformed chemicals and chemocatalytic upgrading of primary chemicals to performance advanced material are presented (highlighted with blue background). Detail of all the chemicals and enzymes can be found in Supplementary Tables 3 and 4.

Production of industrial chemicals, both natural and non-natural, using renewable biomass feedstock via synthetic microbes has been well-established (Chubukov et al., 2016; Cravens et al., 2019; Lee et al., 2019). In the same vein, microbes can be engineered to valorize plastic feedstock, including the PET-derived TPA and EG. Developing advanced and efficient engineered microorganisms to convert and upcycle plastic waste, including PET, is an exciting opportunity for synthetic microbiologists and metabolic engineers. Hence, it is vital to identify the major metabolic and catabolic routes of EG and TPA to develop the microbial chassis for PET upcycling. Both C2 and aromatic metabolic and catabolic pathways are key targets to develop PET upcycle strategies (Kenny et al., 2008). Several microbes capable of EG metabolism has been studied, and among them, *Pseudomonas* is an extensively studied organism. The EG metabolic pathway of *Pseudomonas putida* KT2440 was mapped via comprehensive omics-based systems biology approaches, adaptive laboratory evolution (ALE), and metabolic engineering approaches (Blank et al., 2008; Mückschel et al., 2012; Wehrmann et al., 2017; Franden et al., 2018). Researchers further engineered *Pseudomonas putida* KT2440 for efficient utilization of EG by expression of the entire *gcl* operon and glycolate oxidase (*glcDEF*) operon to overcome the problem of toxic intermediates, glycolaldehyde and glyoxal or knocking out the regulator *glcR* (Franden et al., 2018; Li et al., 2019). Unlike EG, TPA does not freely diffuse via the microbial cell membrane and require a specific TPA transporters. Several TPA transporters and metabolic pathways have been identified and characterized, including the microbes that natively degrade and catabolize PET (Hara et al., 2007; Yoshida et al., 2016; Salvador de Lara et al., 2019; Pardo et al., 2020). Generally, TPA is converted to protocatechuate (PCA) via 1,6-dihydroxycyclohexa-2,4-diene-dicarboxylate (DCD). TphA1A2A3, which is a dioxygenase, catalyzes the conversion of TPA to DCD, and dehydrogenase TphB catalyzes the conversion of DCD to PCA (Frazee et al., 1993; Wang et al., 1995; Maruyama et al., 2004; Kasai et al., 2009; Salvador de Lara et al., 2019). Tph genes in the analysis of databases have revealed similar genetic organization in few organisms belonging to genus *Comamonas*, *Ideonella*, *Ramlibacter*, *Pseudomonas*, and *Rhodococcus* (Choi et al., 2005; Sasoh et al., 2006; Salvador de Lara et al., 2019; Ru et al., 2020). Since we can map the major metabolic and catabolic routes of EG and TPA, we present the overview of potential systematic metabolic engineering routes to efficiently convert PET-derived TPA and EG into high-value chemicals, enabling PET upcycling (Figure 1).

## Engineering Whole-Cell Biocatalysts to Upcycle Plastic

A decade ago, the PET-derived TPA upcycling (obtained from pyrolysis fractionation) into biodegradable plastic polyhydroxyalkanoate (PHA) by *P. putida* (GO16), *P. putida* (GO19), and *Pseudomonas frederiksbergensis* (GO23) has been successfully demonstrated (Kenny et al., 2008). Synthetic microbe-based biological or integrated biological and chemical reactions could be used to produce high-value building blocks, monomers, and fine chemicals obtained by recycled TPA, and EG can then be supplied to the chemical industry. Several models and non-model microbial systems have been developed to upcycle the PET using advanced synthetic microbiology tools in recent years. For instance, EG can be converted to medium chain length polyhydroxyalkanoates (mcl-PHA) using the engineered *P. putida* KT2440, PHAs widely used in many applications, including biomedicine and biodegradable plastic alternative (Franden et al., 2018; Rodriguez-Contreras, 2019). Furthermore, chemical catalytic upgrading could be adopted to convert the PHA into valuable fully deoxygenated hydrocarbon jet (C8–C16)- or diesel (C8–C21)-grade fuels (Linger et al., 2014). Of note, the PHA production of *P. putida* KT2440 could be enhanced by additional strain engineering strategy such as knocking out PHA depolymerase gene *phaZ* and β-oxidation genes *fadBA1* and *fadBA2* and overexpressing *phaG, alkK, phaC1*, and *phaC2* to increase carbon flux into mcl-PHA biosynthesis (Salvachúa et al., 2020). A recent study demonstrated that the hybrid, enzymatic hydrolysis and microbial bioconversion process enables the simultaneous funneling of the PET-derived EG and TPA into PHA and hydroxyalkanoyloxy-alkanoate (HAA), respectively. The evolved strain of *Pseudomonas* sp. GO16 can use both EG and TPA that were used as a biocatalyst and engineered to produce extracellular HAA. The obtained HAA can be converted into a novel biodegradable biopolymer poly(amide urethane) via a chemical catalytic process (Tiso et al., 2020). Kang and coworkers designed a chemo-microbial hybrid process to produce of 2-pyrone-4,6-dicarboxylic acid (PDC), a promising bioplastic monomer from PET-derived TPA (Kang et al., 2020). Engineered *E. coli* consortia were used to produce PDC. One strain was developed by expressing *tphAabc* and *tphB* genes from *Comamonas* sp. E6 to convert TPA into protocatechuic acid (PCA) via 3,4-dihydroxy-cyclohexa-1,5-diene-1,4-dicarboxylic acid (DCD), and the second strain was designed to convert PCA into PDC via 4-carboxy-2-hydroxymuconate semialdehyde by expressing using ligABC genes from *Sphingobium* sp. SYK-6. Another study on upcycling PET degradation monomers describes directing TPA toward the production of high-value aromatics PCA, gallic acid (GA), pyrogallol (PG), catechol (CA), muconic acid (MA), vanillic acid (VA), and EG toward glycolic acid (GLA) (Kim et al., 2019). They engineered *E. coli* strains to harbor relevant metabolic pathways to funnel TPA into the desired product using single or combined reactions of hydroxylation, decarboxylation, oxidative ring cleavage, and methylation. For instance, the above-discussed *Comamonas* sp. *E6* genes were expressed to enable TPA conversion to PCA, and expression of PobA from *P. putida* KT2440 enables hydroxylation of PCA to GA. EG was converted to GLA by EG-fermenting *Gluconobacter oxydans*. Notably, they implemented a tandem reaction approach to improve the production using a double bio-catalytic system. For example, the strain expressing TphAabc and TphB from *Comamonas* sp. E6 was used to convert TPA into PCA, and the strain expressing O-methyltransferase, HsOMT^His^, from *Homo sapiens* was used to convert PCA into VA.

Recently, Rorrer and coworkers demonstrated PET upcycling by incorporating renewably sourced, bio-derived compounds (e.g., muconic acid and acrylic acid) with partially deconstructed PET (i.e., BHET) to manufacture fiberglass-reinforced plastics (Rorrer et al., 2019). *P. putida* KT2440 was engendered to produce innovative chemicals from aromatic catabolism and enables the production of high-performance novel polymers and materials (Johnson et al., 2019). The resulting materials exhibit improved properties relative to the petroleum-based standard (Rorrer et al., 2017; Johnson et al., 2019). For example, as shown in Figure 1, those chemicals could be produced using PET-derived TPA and EG via synthetic microbes.

For instance, β-ketoadipic acid can be obtained from TPA by knocking out the *pcaIJ* in the engineered TPA catabolizing strain (e.g., *tpiABC, tpaAabc*, and *tph*). β-ketoadipic acid can be reacted with hexamethyl diamine (HMDA) to produce a polyamide analogous to nylon 6,6. The polymer showed increased melting temperature and crystallinity and reduced water uptake relative to petroleum-based nylon 6,6 (Sudarsan et al., 2016). It is also possible to funnel EG into 3-hydroxypropionic, and it can be catalytically converted into a high-value acrylic, acrylonitrile, to produce performance advanced polymers and materials (Matsakas et al., 2018). The proposed hybrid biochemical upcycle concept (Figure 1) will enable the end-of-life approach to the PET waste. We anticipated economic incentive from the proposed pathway to produce advanced chemical building blocks. It will remarkably lower the GHG and the energy usage for the production of monomers relative to the fossil-fuel-based production: e.g., upcycling PET into fiberglass-reinforced materials reduces GHG by 40% and energy by 57% (Rorrer et al., 2019).

## Conclusion and Perspectives

Notably, the titer, yield, and rate (TYR) of monomers’ bioproduction from PET-derived substrates (Figure 1) need to be improved via metabolic engineering and process design approaches to enable commercial production. Development of *in silico* computational and machine learning programs to assist the design–build–test–learn cycle (high throughput screening of enzymes and design metabolic pathways) enables rational engineering of commercially applicable superior microbial biocatalyst to upcycle PET. ALE enables the strain to optimize further the engineered genome and fine-tuning of the desired metabolic pathway (Kim et al., 2013; Lee et al., 2014; Oh et al., 2016). We could deploy ALE to improve the PET conversion TYR of the engineered strain.

The waste PET may carry toxic compounds such as emerging contaminants (ECs) and poly organic pollutants (POPs); thus, the process requires *a priori* detoxification steps. For instance, we could use efficient chemical-based metal-organic frameworks or enzyme-based laccase or peroxidase to detoxify the PET-associated ECs and POPs (Pi et al., 2018; Mishra et al., 2019; Morsi et al., 2020). Indeed, laccase can be expressed on PET upcycling microbes to enable *in situ* detoxification (Chen et al., 2016). Given that most of the substrates, intermediates, and targeted products are toxic to the host microbes, engineering multiple toxicity tolerance mechanisms will be necessary. For instance, overcoming the aldehyde tolerance and acid tolerance during EG metabolism could be achieved by alleviating the metabolic bottlenecks and engineering the protein quality control machineries (Franden et al., 2018; Jayakody et al., 2018; Guan and Liu, 2020).

One exciting area of study is adapting the architecture of cellulosomes to develop a multi-enzyme complex capable of efficiently degrading PET. The advanced synthetic biology techniques enable the formulation of large cellulosomes and facilitate superior activity toward the recalcitrant cellulose (Anandharaj et al., 2020). The same concept could be adopted to tailor microbial cell factories to the degradation of high-crystalline PET via developing PETsome (Supplementary Figure 1). It is vital to discover the component that would act as a PET binding domain, analogous to the cellulose-binding domain (Ribitsch et al., 2013; Weber et al., 2019). An efficient cell surface expressing system for bacteria has recently been developed (Chen et al., 2019; Dvořák et al., 2020). Together, those approaches can be implemented to design a consolidated bioprocessing system, a microbial system that can efficiently degrade and upcycle PET into advanced chemicals simultaneously. It will be beneficial to overcome techno-economic challenges such as end-product toxicity on degradation enzymes and the overall operating costs.

In summary, PET upcycling via synthetic microbial biocatalyst or hybrid biochemical approaches has shown great promise to sustainable large-scale solutions for PET waste management in terms of end-of-life to PET. It is essential to perform a comprehensive life cycle and techno-economic analysis to identify the upcycle process’ industrial and environmental feasibility using the engineered biocatalyst. We envision that innovative synthetic microbiology and metabolic engineering approaches may enable the microbial biocatalyst to reach the commercial scale from laboratory bioreactor to upcycle PET, create a circular material economy, and help protect our environment from PET waste.

## Author Contributions

LJ outlined the manuscript. LD surveyed the literature. LJ and LD drafted the article. Both authors read and approved the final version of the manuscript prior to its submission.

## Conflict of Interest

The authors declare that the research was conducted in the absence of any commercial or financial relationships that could be construed as a potential conflict of interest.
