# Supplementary material for: Engineering Microbes to Bio-Upcycle Polyethylene Terephthalate
Source: Front Bioeng Biotechnol. 2021 May 28;9:656465. doi: 10.3389/fbioe.2021.656465 (PMC8193722; doi:10.3389/fbioe.2021.656465)
Supplement: Supplementary file 1 [file Data_Sheet_1.docx]

Supplementary Information

Engineering microbes to bio-upcycle polyethylene terephthalate

Lakshika Dissanayake^1^ and Lahiru N. Jayakody^1, 2*^

^1^School of Biological Science, Southern Illinois University Carbondale, Carbondale, IL, USA.

^2^Fermentation Science Institute, Southern Illinois University Carbondale, Carbondale, IL, USA.

*** Correspondence:** Lahiru Jayakody [lahiru.jayakody@siu.edu](mailto:lahiru.jayakody@siu.edu)

**Table S1 – Overview of PET hydrolyzing enzymes**

| Enzyme | MW  kDa | Optimum | | Isolated  strain | Expression  strain | Tested substrate(s) | Conversion  conditions | products | Reference |
| --- | --- | --- | --- | --- | --- | --- | --- | --- | --- |
|  |  | **T** | **pH** |  |  |  |  |  |  |
| PETase (ISF6_4831) | 28.6 | 30 °C | 9.0 | *Ideonella sakaiensis* 201-F6 | *E.coli* BL21 (DE3) CodonPlus | Low crystallinity PET film | 0.1 mM TPA and 0.2 mM MHET in 18 hours (30 °C, pH 7.0) | TPA, MHET, BHET |  |
|  |  |  |  |  | *Phaeodactylum tricornutum* | Industrially shredded PET | TPA and MHET produced in micromolar quantities (21 °C, pH 8.0) | TPA, MHET | (Moog et al., 2019) |
|  |  |  |  |  | *Chlamydomonas reinhardtii* CC-124 | Powdered commercial beverage PET bottle | 9.12 mg of TPA from 30 mg of PET powder in 4 weeks (30 °C) | BHET, TPA | (Kim et al., 2020) |
|  |  |  |  |  | *Pichia pastoris* | Bis(benzoyloxyethyl) terephthalate | Turnover rate of 1.5 sec^-1^ (30 °C, pH 7.0) | MHET, TPA, BHET | (Chen et al., 2020) |
|  |  |  |  |  |  | Commercial PET bottle | 99 to 109 nM of MHET in 18 hours (30 °C, pH 9.0) |  |  |
| BhrPETase | 27.5 | 80 °C | 6.0-8.0 | *Bacillus subtilis* HR29 | *Bacillus subtilis* strain CBS2 | Amorphous PET powder | More than 6 mM total products in 20 hours (70 °C, pH 8.0) | TPA, MHET, BHET | (Xi et al., 2021) |
| MHETase (ISF6_0224) | 63.1 | 30 °C | 7.0 | *Ideonella sakaiensis* 201-F6 | *E. coli* Rosetta-gami B(DE3) | MHET | MHET turnover rate of 31 ± 0.8 s−1 | TPA, EG | (Yoshida et al., 2016) |
| Ple611  Ple628  Ple629  Ple200  Ple201  Ple453 | NA | 30 °C | 7.0 | Marine microbial consortium | NA | PBAT-based blend film (PF) | Highest degradation within 15 days (11-16% mineralized per day) (22 °C, pH 7.0) | ADPA, TPA, SA, TB | (Meyer-Cifuentes et al., 2020) |
| Tcur1278 | 35 | 60 °C | 8.5 | *Thermomonospora curvata* DSM43183 | Escherichia coli TOP10 | PET nanoparticles | Maximum hydrolysis rate of 3.3 × 10^-3^ min^-1^ with 80 μg/mL of enzyme in 60 minutes (50 °C, pH 8.5) | NA | (Wei et al., 2014) |
| Tcur0390 | 35 | 55 °C | 8.5 | *Thermomonospora curvata* DSM43183 | Escherichia coli TOP10 | PET nanoparticles | Maximum hydrolysis rate of 5.9 × 10^-3^ min^-1^ with 20 μg/mL of enzyme in 60 minutes (50 °C, pH 8.5) | NA | (Wei et al., 2014) |
| PET2 (lipIAF5-2) | NA | 70 °C | 8.0 – 9.0 | Uncultured bacterium | E. coli T7 | Low-crystallinity PET film | 900 μM terephthalic acid with 100 μg of PET 2 after 24 h of incubation (60 °C, pH 7.5) | TPA | (Danso et al., 2018) |
| PET5 (lipA OLEAN_C07960) | NA | NA | NA | *Oleispira antarctica* RB-8 | E. coli T7 | Low-crystallinity PET film | Halos in agar plates containing PET nanoparticles after overnight incubation | NA | (Danso et al., 2018) |
| PET6 | NA | 55 °C | 8.0 – 9.0 | *Vibrio gazogenes* | E. coli T7 | Low-crystallinity PET film | Halos in agar plates containing PET nanoparticles after overnight incubation | NA | (Danso et al., 2018) |
| PET12 (AAW51_2473) | NA | NA | NA | *Polyangium brachysporum* | E. coli T7 | Low-crystallinity PET film | Halos in agar plates containing PET nanoparticles after overnight incubation | NA | (Danso et al., 2018) |
| PE-H | 32 | NA | NA | *Pseudomonas aestusnigri*VGXO14^T^ | *E. coli* BL21(DE3) | Amorphous PET film | 4 mg/l MHET produced within 48 hours (30 °C, pH 7.4) | MHET | (Molitor et al., 2020) (Bollinger et al., 2020) |
| HiC | NA | 70-  80 °C |  | *Humicola insolens* | NA | 7% crystalline PET film | 97±3% weight loss in 96 hours (70 °C, pH 8.0) | TPA | (Ronkvist et al., 2009) |
| PmC | NA |  |  | *Pseudomonas mendocina* | NA | 7% crystalline PET film | 5% weight loss in 96 hours (50 °C, pH 8.0) | TPA | (Ronkvist et al., 2009) |
| FsC | NA |  |  | *Fusarium solani* | NA | 7% crystalline PET film | 5% weight loss in 96 hours (40 °C, pH 8.0) | TPA | (Ronkvist et al., 2009) |
| Thc_Cut1 | 29.4 | 50°C | 7.0 | *Thermobifida cellulosilytica* DSM44535 | *Escherichia coli* BL21-Gold (DE3) | 37% crystalline PET film | More than 50mmol TPA and less than 10mmol MHET per mol of enzyme in 72 hours (50°C, pH 7.0) | TPA, MHET  TPA, MHET | (Herrero Acero et al., 2011) |
|  |  |  |  |  | *Pichia pastoris* | 24% crystalline PET powder | More than 50mM soluble released products from 5µM enzyme in 96 hours (65°C, pH 8.0) |  | (Gamerith et al., 2017) |
| Thc_Cut2 | 29.7 | 50°C | 7.0 | *Thermobifida cellulosilytica* DSM44535 | *Escherichia coli* BL21-Gold (DE3) | 37% crystalline PET film | Less than 10mmol TPA and more than 10mmol MHET per mol of enzyme in 72 hours (50°C, pH 7.0) | TPA, MHET | (Herrero Acero et al., 2011) |
| Thf42_Cut1 | 29.6 | NA | NA | *Thermobifida* *fusca* DSM44342 | *Escherichia coli* BL21-Gold (DE3) | 37% crystalline PET film | More than 40mmol TPA and less than 10mmol MHET per mol of enzyme in 72 hours (50°C, pH 7.0) | TPA, MHET | (Herrero Acero et al., 2011) |
| Tha_Cut1 | 28.1 | NA | NA | *Thermobifida alba* | *Escherichia coli* BL21-Gold (DE3) | Bis(benzoyloxyethyl) terephthalate (3PET) | More than 15mmol TPA and more than 30mmol MHET per mol of enzyme in 2 hours (50°C, pH 7.0) | TPA, MHET, BA, HEB | (Ribitsch et al., 2012a) |
| Cut190 | 30.3 | 65– 75 °C | 6.0 – 8.5 | *Saccharomonospora viridis* AHK190 | *Escherichia coli* Rosetta-gami B (DE3) | NA | Hydrophilzed PET film overnight (50°C) | NA | (Kawai et al., 2014) |
| LCC | 28 | 50-70°C | 8.0 | Leaf branch compost | *E. coli* BL21-CodonPlus(DE3) | Amorphous PET film | ≤ 25% weight loss in 24 hours (70°C, pH 8.5) | TPA, EG | (Sulaiman et al., 2012) |
| Thh_Est | NA | NA | NA | *Thermobifida halotolerans* DSM44931 | *E. coli* BL21-Gold (DE3) | Bis(benzoyloxyethyl) terephthalate (3PET) | More than 15mmol TPA and more than 30mmol MHET per mol of enzyme in 2 hours (50°C, pH 7.0) | TPA, MHET, BA, HEB | (Ribitsch et al., 2012b) |
| BsEstB | NA | 40°C | 7.0 | *Bacillus subtilis* | *Escherichia coli* BL21-Gold (DE3) | Bis(benzoyloxyethyl) terephthalate (3PET) | 800 µg TPA in 36 hours (30°C, pH 7.0) | TPA, MHET, BA, HEB | (Ribitsch et al., 2011) |
| TfH | 28 | 65 – 70 °C | 6.0 – 7.0 | Thermobifida fusca DSM43793 | *E.coli*  *Escherichia coli* BL21(DE3) | Amorphous PET film  Semi crystalline PET film | 50% weight loss in 3 weeks (55°C, pH 7.0)  ≤ 14% weight loss in the presence of 10 mM CaCl_2_ in 48 hours (65 °C, pH 8.5) | TPA, EG | (Kleeberg et al., 2005;Müller et al., 2005)  (Then et al., 2015) |
| Est119 | 30 | 50 °C | 6.0 | Thermobifida alba AHK119 | *Escherichia coli* Rosetta-gami B (DE3) | Poly(caprolactone) (PCL) | TPA acid detected by HPLC (50 °C, pH 7.0) | TPA | (Hu et al., 2008;Thumarat et al., 2012) |
| TfCut1 | NA | 55-65 °C | 8.5 | Thermobifida fuscaKW3 | *Escherichia coli* BL21(DE3) | Semi crystalline PET film | ≤ 11% weight loss in the presence of 10 mM CaCl_2_ in 48 hours (65 °C, pH 8.5) | NA | (Then et al., 2015) |
| TfCut2 | 30.8 | 55-65 °C | 8.5 | Thermobifida fusca KW3 | *Escherichia coli* BL21(DE3) | Semi crystalline PET film | ≤ 12% weight loss in the presence of 10 mM CaCl_2_ in 48 hours (65 °C, pH 8.5) | NA | (Roth et al., 2014;Then et al., 2015) |
| Cbotu_EstA | NA | 50 – 60 °C | 7.0 | Clostridium botulinumATCC3502 | *E. coli* BL21-Gold (DE3) | PET film | ≤ 5 U^-1^ mol enzyme in 72 hours (50 °C, pH 7.0) | MHET | (Biundo et al., 2018) |
| BTA-2 | 32.5 | 50 °C | 8.5 | *Thermobifida fusca* DSM43793 | *Escherichia coli* BL21(DE3) | Semi crystalline PET film | ≤ 4% weight loss in the presence of 10 mM CaCl_2_ in 48 hours (65 °C, pH 8.5) | NA | (Kleeberg et al., 2005;Müller et al., 2005;Then et al., 2015) |
| Tfu_0882 | 34.4 | 55 - 65 °C | 8.0 | *Thermobifida fusca*YX (*T. fusca* WSH03-11) | *Escherichia coli* BL21(DE3) | Semi crystalline PET film | ≤ 5% weight loss in the presence of 10 mM CaCl_2_ in 48 hours (65 °C, pH 8.5) | NA | (Lykidis et al., 2007;Chen et al., 2008;Su et al., 2013;Then et al., 2015) |
| Tfu_0883 | 32.2 | 60 °C | 8.0 | *Thermobifida fusca*YX (*T. fusca* WSH03-11) | Escherichia coli JM109 | NA | NA | NA | (Lykidis et al., 2007;Chen et al., 2008;Su et al., 2013) |
| TfAXE | 28 | 60 °C | 7.5 | *Thermobifida fusca*NTU22 | E. coli DH5α | NA | NA | NA | (Huang et al., 2010) |
| Cut1 | 30.1 | 55 °C | 8.0 | *Thermobifida fusca*NRRL B-8184 | *Escherichia coli* BL21 (DE3) | NA | NA | NA | (Hegde and Dasu, 2013) |
| Cut2 | 29.6 | 55 °C | 8.0 | *Thermobifida fusca*NRRL B-8184 | *Escherichia coli* BL21 (DE3) | NA | NA | NA | (Hegde and Dasu, 2013) |

TPA – Terephthalic acid, EG – Ethylene glycol, MHET - mono-2-hydroxyethyl terephthalate, HEB - 2-hydroxyethyl benzoate BA - Benzoic acid, TB – Terephthalate butanediol monoester, SA – Sebacic acid, ADPA – Adipic acid, NA – Data not available

**Table S2 – Engineered PET hydrolyzing enzymes**

| **Original Enzyme** | **Mutations** | **Host strain** | **Expression vector** | **Affinity tag** | **Tested**  **Substrate(s)** | **PET conversion & conditions** | **Reference** |
| --- | --- | --- | --- | --- | --- | --- | --- |
| PETase | S121E/D186H/R280A | *E. coli* strain  Rosetta gami - B | pET15b | N-terminal His_6_-tag | Commercial PET film | 83.3 μm MHET and 37.6 μm TPA within 72 hours (40 °C, pH 9.0) | (Son et al., 2019) |
| PETase | S214H-I168R-W159H-S188Q-R280A-A180I-G165A-Q119Y-L117F-T140D | *E. coli* TOP10 | pET21b-PETase | C-terminal His_6_-tag | Semicrystalline PET film | More than 2.5 mM total compounds released in 10 days (37 °C, pH 9.0) | (Cui et al., 2019) |
| PETase | R280A | *E. coli* Rosetta gami-B | pET15b | C-terminal His_6_-tag | BHET  Commercial PET film | Similar activity to wild type PETase (30 °C, pH 7.0)  Increased activity by 22.4% relative to wild type PETase (30 °C, pH 9.0) | (Joo et al., 2018) |
| PETase | S238F/W159H | *E. coli* C41(DE3) | pET- 21b(+) | C-terminal His_6_-tag | PET coupons with an initial crystallinity of 14.8 ± 0.2% | 4.13% higher crystallinity loss in 96 hours (30 °C, pH 7.2) | (Austin et al., 2018) |
| PETase | I179F | E. coli BL21 (DE3) | pET28a | His tag | PET film | 6 mmol.^L-1^ TPA in 48 hours, degradation rate of 22.5 mg per μmol·L^−1^  (30 °C, pH 8.5) | (Ma et al., 2018) |
| PETase | Y58A | *E. coli* BL21-CodonPlus (DE3) RIPL | pET-21b | C-terminal His_6_-tag | Commercial PET drinking bottle | More than 30 nM MHET and more than 20 nM TPA in 20 hours (30 °C, pH 9.0) | (Liu et al., 2018) |
| PETase | R53E | *E. coli* BL21-CodonPlus (DE3) | pET-21b | C-terminal His_6_-tag | Low crystallinity PET | More than 0.2 nmol min^-1^cm^-2^ (30 °C, pH 8.0 & 9.0) | (Furukawa et al., 2018) |
| PETase | Y58A  T59A | *E. coli* BL21trxB (DE3) | pET32a | His tag | PET film | More than 80% and 90% MHET production respectively in 42 hours (30 °C, pH 9.0) | (Han et al., 2017) |
| MHETase | R411K/S416A/F424I | *E. coli* BL21 (DE3) -T1R | pET22b (+) | C-terminal His_6_-tag | Amorphous PET film | Enzyme activity of 8 μm in 72 hours (30 °C, pH 8.0) | (Sagong et al., 2020) |
| MHETase | S416A_F424N  R411A_S419G_F424N  W397A | *E. coli* Shuffle T7 | pColdII | C-terminal His_6_-tag | BHET | More than 0.12 s^-1^ turnover rate of BHET.  More than 20 s^-1^ turnover rate of MHET with W397A (30 °C, pH 7.5) | (Palm et al., 2019) |
| LCC | LCC variant | *E. coli* BL21 (DE3) | pET26b | C-terminal His_6_-tag | Amorphized bottle grade PET | 90% PET depolymerization in 10 hours (72 °C, pH 8.0) | (Tournier et al., 2020) |
| LCC | DSM1313::pHK‐LCC * | *Clostridium thermocellum* | pHK | NA | Amorphous PET film | 62% weight loss in 14 days (60 °C, pH 7.4) | (Yan et al., 2020) |
| LCC | LCC-G | *Pichia pastoris* | PET28 | NA | 7% crystalline PET film | ∼95% weight loss in 48 hours (70 °C, pH 8) | (Herrero Acero et al., 2011;Shirke et al., 2018) |
| Thc_Cut2 | Thc_Cut2 variant | *Eschericha coli* BL21- Gold (DE3) | pET26b(+) | C-terminal His_6_-tag | PET film | ≤ 0.45 mM TPA in 2 days (50 °C, pH 7.4) | (Herrero Acero et al., 2013) |
| Thc_Cut1 | Thc_Cut1_ko_Asn | *Pichia pastoris* | pPICZαB | C-terminal His_6_-tag | 24% crystalline PET powder | More than 60mM soluble released products from 5µM enzyme in 96 hours (65°C, pH 8.0) | (Gamerith et al., 2017) |
| Thc_Cut1 | Thc_Cut1_ko_ST | *Pichia pastoris* | pPICZαB | C-terminal His_6_-tag | 24% crystalline PET powder | More than 60mM soluble released products from 5µM enzyme in 96 hours (65°C, pH 8.0) | (Gamerith et al., 2017) |
| Est119 | A68V/S219P | *Eschericha coli* Roseta-gami B (DE3) | pQE80L | N-terminal His_6_-tag | p-nitrophenyl butyrate | 50-fold increase of activity over the wild type  (37 °C, pH 7.0) | (Thumarat et al., 2012) |
| Tfu_0883 | Q132A/T101A | NA | pET20b | NA | PET fabric | 390 mM TPA produced in 48 hours (60 °C, pH 7.5) | (Silva et al., 2011) |
| TfH | rTfH** | Escherichia coli TG1 (DSM 6056) | pCytexP1-OmpA-bta1 | C-terminal His_6_ tag | NA | NA | (Dresler et al., 2006) |
| Cut190 | S226P/R228S | *Escherichia coli* Rosetta-gami B (DE3) | pQE8oL | His_6_ tag | Amorphous PET  PET film from PET package | Degradation rate of 13.5 ± 0.5 % in 72 hours (63°C, pH 8.2)  Degradation rate of 27.0 ± 1.0 % in 72 hours (63°C, pH 8.2) | (Kawai et al., 2014) |
| Cut190 | Cut190*/Q138A  /D250C-E296C | NA | NA | NA | PET microfiber | 24.8% degradation in 113 hours (30 °C, pH 8.5) | (Kawai et al., 2019) |
| Cut190 | Q138A/D250C-E296C  /Q123H/N202H | *Escherichia coli* Rosetta-gami B (DE3) | NA | His_6_ tag | Microfiber amorphous PET | More than 30% degradation rate in 3 days (70 °C, pH 8.5) | (Oda et al., 2018) |
| Cut190 | I224A/ Q138A | *Escherichia coli* Rosetta-gami B (DE3) | NA | N-terminal His_6_-tag | BHET | Catalytic activity of 150±0.2 s^-1^  (37 °C, pH 8.2) | (Kawabata et al., 2017) |
| PE-H | Y250S | E. coli DH5α | pET22b_PE-H_c6H_ | His_6_ tag | Amorphous PET film | More than 5 mg/l MHET in 48 h (30 °C, pH 7.4) | (Bollinger et al., 2020) |
| TfCut2 | G62A/F209A | *E. coli* BL2 (DE3) | pET21-b | C-terminal His_6_ tag | Low crystallinity PET film | Degradation rate of 97 ± 1.8% within 30 hours (65 °C, pH 9.0) | (Furukawa et al., 2019) |
| TfCut2 | G62A/I213S | *E. coli* BL2 (DE3) | NA | NA | Amorphous PET film | ≤ 45% weight loss after 50 h  (65 °C, pH 8.0) | (Wei et al., 2016) |
| TfCut2 | D204C-E253C(γ), γ -D174L, γ-D174N, γ-D174R, γ-D174A, γ-D174R-G205D | *E. coli* BL2 (DE3) | NA | NA | Amorphous PET film | ≤ 25% weight loss after 48 h  (65 - 80 °C, pH 8.0) | (Then et al., 2016) |
| Cbotu_EstA | Del171_Cbotu_EstA_S119A | *E. coli* BL21-Gold (DE3) | NA | NA | PET film | ≤ 5 U^-1^ mol enzyme in 72 hours (50 °C, pH 7.0) | (Biundo et al., 2018) |

* Signal peptide sequence of Cel48S used for secretory production of LCC

** Express as a fusion protein using the OmpA leader sequence and a His_6_ tag

TPA- Terephthalic acid, MHET – Monohydroxyethyl terephthalate, BHET – Bis (2-hydroxyethyl terephthalate), NA- Data not available

| **Table S3 – Compound list of Figure 1** |
| --- |

| Number | Compound |
| --- | --- |
| 1 | Polyethylene terephthalate |
| 2 | Bis(2-Hydroxyethyl) terephthalate |
| 3 | Ethylene glycol |
| 4  5 | Terephthalic acid  Sodium terephthalate |
| 6 | (3*S*,4*R*)-3,4-Dihydroxy-1,5-cyclohexadiene-1,4-dicarboxylic acid |
| 7 | Protocatechuate |
| 8 | (*Z*)-(*E*)-4-Formylmethylidene-2-hydroxy-2-pentenedioate |
| 9 | 4-Carboxy-2-hydroxymuconate-semialdehyde |
| 10 | 2-Pyrone-4,6-dicarboxylic acid |
| 11 | 4-Oxalomesaconic acid (enol form) |
| 12 | 4-Oxalomesaconic acid (Keto form) |
| 13 | 4-carboxy-4-hydroxy-2-oxoadipic acid |
| 14 | Pyruvate |
| 15 | β-Carboxy-cic,cis-mucinic acid |
| 16 | ϒ-Carboxymuconolactone |
| 17 | Muconolactone |
| 18 | β-Ketoadipic acid |
| 19 | 3-oxoadipyl-CoA |
| 20 | Succinyl-CoA |
| 21 | Acetyl-CoA |
| 22 | 5-Carboxy-2-hydroxymuconate-semialdehyde |
| 23 | 2-Hydroxymuconate-semialdehyde |
| 24 | 4-Oxalocrotonic acid (enol form) |
| 25 | 4-Oxalocrotonic acid (keto form) |
| 26 | 4-Hydroxy-2-oxovaleric acid/2-Oxo-4-pentenoate |
| 27 | 4-Hydroxy-2-oxovaleric acid |
| 28 | Acetaldehyde |
| 29 | Glycolaldehyde |
| 30 | Glycolate |
| 31 | Glyoxalate |
| 32 | Tartronate semialdehyde |
| 33 | Hydroxypyruvate |
| 34 | Glycerate |
| 35 | 2-phosphoglycerate |
| 36 | Phosphoenolpyruvate |
| 37 | Gallic acid |
| 38 | Pyrogallol |
| 39 | Vanillin |
| 40 | Catechol |
| 41 | *cis,cis*-muconate |
| 42 | Malonyl-CoA |
| 43 | Malonyl-ACP |
| 44 | Acetoacyl-ACP |
| 45 | 3-Ketoacyl-ACP |
| 46 | (R)-3-Hydroxyacyl-ACP |
| 47 | Enoyl-ACP |
| 48 | Acyl-ACP |
| 49 | Malondialdehyde |
| 50 | 3-Hydroxypropionic acid |
| 51 | (R)-3-Hydroxyfatty acid |
| 52 | (R)-3-Hydroxyacyl-CoA |
| 53 | Medium chain length polyhydroxyalkanoate (mcl-PHA) |
| 54 | 2-Trans-Enoyl--CoA |
| 55 | Citrate |
| 56 | Isocitrate |
| 57 | α-Ketoglutarate |
| 58 | Succinyl-CoA |
| 59 | Fumarate |
| 60 | Malate |
| 61 | Oxaloacetate |
| 62 | Adipic acid |

**Table S4: Description of enzymes of the biofunneling pathways represent in Figure 01**

| Protein | Enzyme name | EIC number |
| --- | --- | --- |
| AceE | Pyruvate dehydrogenase E1 component | EC:1.2.4.1 |
| AcnA | Aconitate hydratase | EC:4.2.1.3 |
| AlkK | Acyl-CoA synthetase | EC:6.2.1.3 |
| AroY | Protocatechuate decarboxylase | EC:4.1.1.68 |
| CatA | Catechol 1,2-dioxygenase | EC:1.13.11.1 |
| CatBC | Muconate cycloisomerase 1/Muconolactone Delta-isomerase | EC:5.5.1.1/EC:5.3.3.4 |
| Eno | Enolase | EC:4.2.1.11 |
| ER | Enoate reductase | EC:1.3.1.31 |
| AccA | Acetyl-CoA carboxylase | EC:6.4.1.2 |
| FabAZ | 3-hydroxydecanoyl-[acyl-carrier-protein] dehydratase/3-hydroxyacyl-[acyl-carrier-protein] dehydratase FabZ | EC:4.2.1.59 |
| FabBF | 3-oxoacyl-[acyl-carrier-protein] synthase 1/3-oxoacyl-[acyl-carrier-protein] synthase 2 | EC:2.3.1.41/EC:2.3.1.179 |
| FabD | Malonyl CoA-acyl carrier protein transacylase | EC:2.3.1.39 |
| FabG | 3-oxoacyl-[acyl-carrier-protein] reductase | EC:1.1.1.100 |
| FabH | 3-oxoacyl-ACP synthase | EC:2.3.1.180 |
| FabIV | Enoyl-[acyl-carrier-protein] reductase [NADH] | EC:1.3.1.9 |
| GalB | 4-oxalmesaconate hydratase | EC:4.2.1.83 |
| GalC | 4-carboxy-4-hydroxy-2-oxoadipic acid aldolase | EC:4.1.3.17 |
| GalD | 4-oxalomesaconate tautomerase | EC:5.3.2.8 |
| Gcl | Glyoxylate carboligase | EC:4.1.1.47 |
| GlcDEF | Glycolate oxidase, putative FAD-linked subunit/Glycolate oxidase, putative FAD-binding subunit/ | EC:1.1.99.14 |
| gltA | Citrate synthase | EC:2.3.3.16 |
| GlxR | Tartronate semialdehyde reductase | EC:1.1.1.60 |
| HsoMT | Catechol O-methyltransferase | EC:2.1.1.6 |
| Hyi | Hydroxypyruvate isomerase | EC:5.3.1.22 |
| lcd | Isocitrate dehydrogenase [NADP] | EC:1.1.1.42 |
| LigAB | Type II extradiol dioxygenases/ protocatechuate 4,5-dioxygenase | [EC:1.13.11.](https://enzyme.expasy.org/EC/6.5.1.2)8 |
| LigC | 4-carboxy-2-hydroxymuconate-6-semialdehyde dehydrogenase | EC:1.1.1.312 |
| LigI | 2-pyrone-4,6-dicarboxylate hydrolase | EC:3.1.1.57 |
| Lpdc | Gallate decarboxylase | EC:4.1.1.59 |
| Mcr | malonyl-CoA reductase | EC 1.1.1.298 |
| Mdh | Probable malate dehydrogenase | EC:1.1.1.37 |
| PcaB | 3-carboxy-cis,cis-muconate cycloisomerase | EC:5.5.1.2 |
| PcaC | 4-carboxymuconolactone decarboxylase | EC:4.1.1.44 |
| PcaD | 3-oxoadipate enol-lactonase 2 | EC:3.1.1.24 |
| PcaF | 3-oxoadipyl-CoA thiolase | EC:2.3.1.174 |
| PcaHG | Protocatechuate 3,4-dioxygenase beta chain/Protocatechuate 3,4-dioxygenase alpha chain | EC:1.13.11.3 |
| PcaIJ | 3-oxoadipate CoA-transferase | EC:2.8.3.6 |
| PedEH | PQQ-dependent dehydrogenase | EC:1.1.2.8 |
| PedI | Aldehyde dehydrogenase | EC:1.2.1.3 |
| PhaC | Poly(3-hydroxyalkanoate) polymerase 2 | EC:2.3.1.- |
| PhaG | (R)-3-hydroxydecanoyl-ACP:CoA transacylase | EC:2.4.1.- |
| PhaJ | (R)-specific enoyl-CoA hydratase | EC:4.2.1.119 |
| AceA-D | Isocitrase | EC:4.1.3.1 |
| PobA | p-hydroxybenzoate hydroxylase | EC:1.14.13.2 |
| PP_0897 | Fumarate hydratase class I | EC:4.2.1.2 |
| PP_4300 | Putative hydroxypyruvate reductase | EC:1.1.1.81 |
| PraA | Protocatechuate 2,3-dioxygenase | EC:1.13.11.8 |
| PraH | 5-carboxy-2-hydroxymuconate-6-semialdehyde decarboxylase | EC:[4.1.1.45](https://www.genome.jp/dbget-bin/www_bget?ec:4.1.1.45) |
| PykAF | Pyruvate kinase | EC:2.7.1.40 |
| SdhB | Succinate dehydrogenase iron-sulfur subunit | EC:1.3.5.1 |
| SucAB | Oxoglutarate dehydrogenase (succinyl-transferring)/Dihydrolipoyllysine-residue succinyltransferase component of 2-oxoglutarate dehydrogenase complex | EC:1.2.4.2/ EC:2.3.1.61 |
| SucCD | Succinate--CoA ligase [ADP-forming] subunit beta/ | EC:6.2.1.5 |
| TphAabc | Terephthalate 1,2-dioxygenase | EC:1.14.12.15 |
| TphB | 4-hydroxythreonine-4-phosphate dehydrogenase | EC:1.1.1.262 |
| TpiABC | Triosephosphate isomerase/Small transmembrane protein of the aromatic acids transporter | EC:5.3.1.1 |
| XylG | 2-hydroxymuconic semialdehyde dehydrogenase | EC:1.2.1.85 |
| XylH | 2-hydroxymuconate tautomerase | EC:5.3.2.6 |
| XylI | 4-oxalocrotonate decarboxylase | EC:4.1.1.77 |
| XylJ | 2-oxopent-4-enoate hydratase | EC:4.2.1.80 |
| XylK | 4-hydroxy-2-oxovalerate aldolase | EC:4.1.3.39 |
| XylQ | Acetaldehyde dehydrogenase | EC:1.2.1.10 |


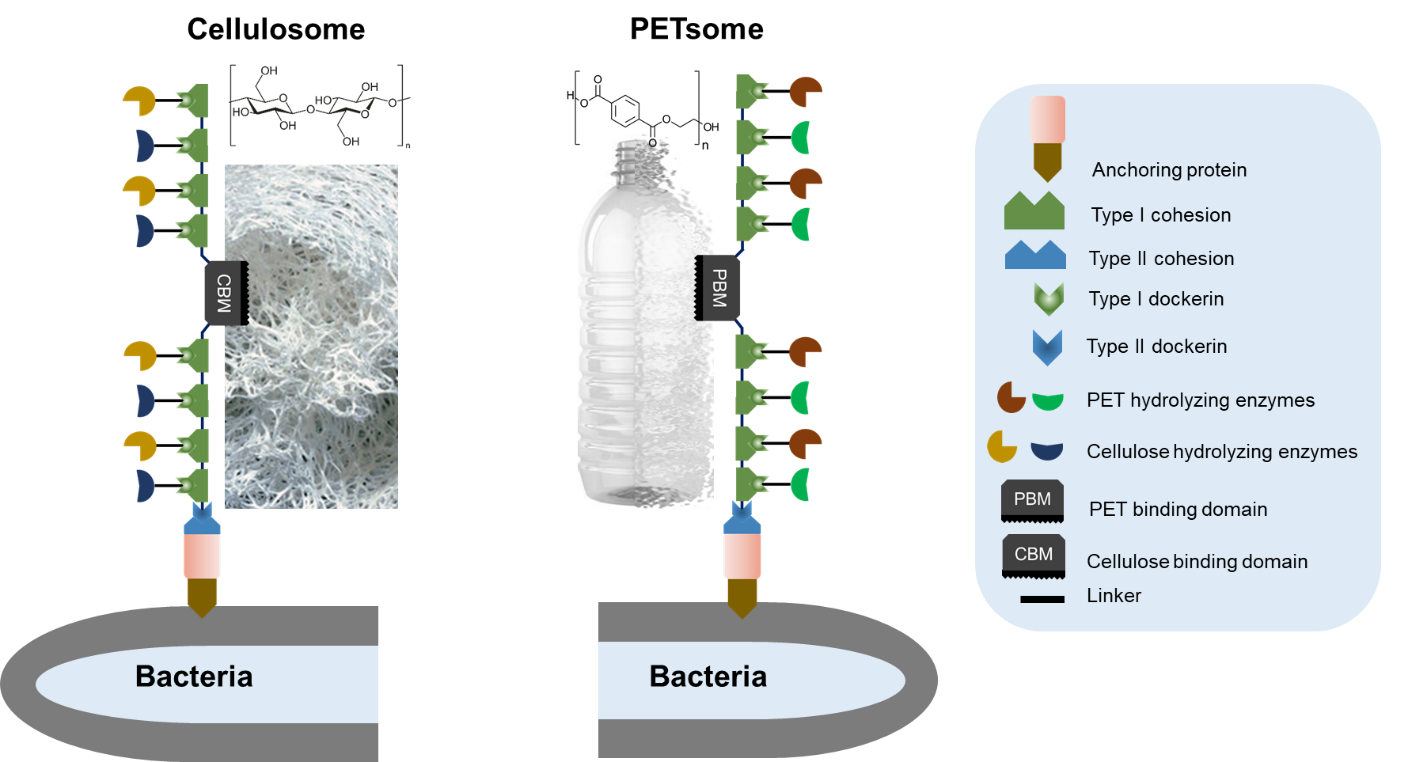


**Figure S1:** Architecture of the proposed PETsome**.**

Most of the cellulosome's key components can be copied to construct PETsome, including cohesion, dockerin domains, the linkers (27 – 35 amino acid long flexible linkers), and the anchoring protein (Krasteva et al., 2017;Anandharaj et al., 2020;Dvořák et al., 2020b;Vita et al., 2020). Contrary to the cellulosome, the PET binding domain and PET hydrolyzing enzymes need to be engineered to form the PETsome. PETsome can be assembled either in vitro using purified components or in vivo on a suitable microbial host's surface.

The strategies suggested by Dvořák and coworker can be adopted to develop an efficient PETsome on *P. putida*, a popular workhorse for plastic upcycling (Dvořák et al., 2020b). They highlighted that surface engineering for removing non-essential outer membrane structures (fimbriae, pili, curli, adhesins, exopolysaccharides, and lipopolysaccharides) *P. putida* (i.e., *P. putida* EM71) enhances *in vivo* cellulosome formation relative to the parental strain. They used monomeric type V secretion pathway protein, Ag43 autotransporters to cell surface display of PETsome. The Ag43 gene encodes all three domains needed for display (a signal peptide, a surface-exposed passenger, and a transmembrane β-domain).

**References:**

Anandharaj, M., Lin, Y.J., Rani, R.P., Nadendla, E.K., Ho, M.C., Huang, C.C., Cheng, J.F., Chang, J.J., and Li, W.H. (2020). Constructing a yeast to express the largest cellulosome complex on the cell surface. *Proceedings of the National Academy of Sciences* 117**,** 2385. doi:10.1073/pnas.1916529117.

Austin, H.P., Allen, M.D., Donohoe, B.S., Rorrer, N.A., Kearns, F.L., Silveira, R.L., Pollard, B.C., Dominick, G., Duman, R., El Omari, K., Mykhaylyk, V., Wagner, A., Michener, W.E., Amore, A., Skaf, M.S., Crowley, M.F., Thorne, A.W., Johnson, C.W., Woodcock, H.L., Mcgeehan, J.E., and Beckham, G.T. (2018). Characterization and engineering of a plastic-degrading aromatic polyesterase *Proceedings of the National Academy of Sciences* 115**,** E4350. doi:10.1073/pnas.1718804115.

Biundo, A., Reich, J., Ribitsch, D., and Guebitz, G.M. (2018). Synergistic effect of mutagenesis and truncation to improve a polyesterase from *Clostridium botulinum* for polyester hydrolysis. *Scientific Reports* 8**,** 3745. doi:10.1038/s41598-018-21825-9.

Bollinger, A., Thies, S., Knieps-Grünhagen, E., Gertzen, C., Kobus, S., Höppner, A., Ferrer, M., Gohlke, H., Smits, S.H.J., and Jaeger, K.-E. (2020). A novel polyester hydrolase from the marine bacterium *Pseudomonas aestusnigri* - structural and functional insights. *Frontiers in microbiology* 11**,** 114-114. doi:10.3389/fmicb.2020.00114.

Chen, S., Tong, X., Woodard, R.W., Du, G., Wu, J., and Chen, J. (2008). Identification and characterization of bacterial cutinase. *The Journal of biological chemistry* 283**,** 25854-25862. doi:10.1074/jbc.M800848200.

Chen, Z., Wang, Y., Cheng, Y., Wang, X., Tong, S., Yang, H., and Wang, Z. (2020). Efficient biodegradation of highly crystallized polyethylene terephthalate through cell surface display of bacterial PETase. *Sci Total Environ* 709**,** 136138. doi:10.1016/j.scitotenv.2019.136138.

Cui, Y., Chen, Y., Liu, X., Dong, S., Tian, Y.E., Qiao, Y., Mitra, R., Han, J., Li, C., Han, X., Liu, W., Chen, Q., Du, W., Tang, S., Xiang, H., Liu, H., and Wu, B. (2019). Computational redesign of a PETase for plastic biodegradation by the GRAPE strategy *bioRxiv***,** 787069. doi:10.1101/787069.

Danso, D., Schmeisser, C., Chow, J., Zimmermann, W., Wei, R., Leggewie, C., Li, X., Hazen, T., and Streit, W.R. (2018). New insights into the function and global distribution of polyethylene terephthalate (PET)-degrading bacteria and enzymes in marine and terrestrial metagenomes. *Applied and Environmental Microbiology* 84**,** e02773-02717. doi:10.1128/AEM.02773-17.

Dresler, K., Van Den Heuvel, J., Müller, R.-J., and Deckwer, W.-D. (2006). Production of a recombinant polyester-cleaving hydrolase from *Thermobifida fusca* in *Escherichia coli*. *Bioprocess and biosystems engineering* 29**,** 169-183. doi:10.1007/s00449-006-0069-9.

Dvořák, P., Bayer, E.A., and De Lorenzo, V. (2020). Surface display of designer protein scaffolds on genome-reduced strains of *Pseudomonas putida*. *bioRxiv***,** 2020.2005.2013.093500. doi:10.1101/2020.05.13.093500.

Furukawa, M., Kawakami, N., Oda, K., and Miyamoto, K. (2018). Acceleration of enzymatic degradation of poly(ethylene terephthalate) by surface coating with anionic surfactants. *ChemSusChem* 11**,** 4018-4025. doi:<https://doi.org/10.1002/cssc.201802096>.

Furukawa, M., Kawakami, N., Tomizawa, A., and Miyamoto, K. (2019). Efficient degradation of poly(ethylene terephthalate) with *Thermobifida fusca* cutinase exhibiting improved catalytic activity generated using mutagenesis and additive-based approaches. *Scientific Reports* 9**,** 16038. doi:10.1038/s41598-019-52379-z.

Gamerith, C., Vastano, M., Ghorbanpour, S.M., Zitzenbacher, S., Ribitsch, D., Zumstein, M.T., Sander, M., Herrero Acero, E., Pellis, A., and Guebitz, G.M. (2017). Enzymatic degradation of aromatic and aliphatic polyesters by *P. pastoris* expressed cutinase 1 from *Thermobifida cellulosilytica*. *Frontiers in Microbiology* 8**,** 938.

Han, X., Liu, W., Huang, J.-W., Ma, J., Zheng, Y., Ko, T.-P., Xu, L., Cheng, Y.-S., Chen, C.-C., and Guo, R.-T. (2017). Structural insight into catalytic mechanism of PET hydrolase *Nature Communications* 8**,** 2106. doi:10.1038/s41467-017-02255-z.

Hegde, K., and Dasu, V. (2013). Production optimization and characterization of recombinant cutinases from *Thermobifida fusca* sp. NRRL B-8184 *Applied biochemistry and biotechnology* 170. doi:10.1007/s12010-013-0219-x.

Herrero Acero, E., Ribitsch, D., Dellacher, A., Zitzenbacher, S., Marold, A., Steinkellner, G., Gruber, K., Schwab, H., and Guebitz, G.M. (2013). Surface engineering of a cutinase from *Thermobifida cellulosilytica* for improved polyester hydrolysis. *Biotechnology and Bioengineering* 110**,** 2581-2590. doi:<https://doi.org/10.1002/bit.24930>.

Herrero Acero, E., Ribitsch, D., Steinkellner, G., Gruber, K., Greimel, K., Eiteljoerg, I., Trotscha, E., Wei, R., Zimmermann, W., Zinn, M., Cavaco-Paulo, A., Freddi, G., Schwab, H., and Guebitz, G. (2011). Enzymatic surface hydrolysis of PET: Effect of structural diversity on kinetic properties of cutinases from *Thermobifida* *Macromolecules* 44**,** 4632-4640. doi:10.1021/ma200949p.

Hu, X., Osaki, S., Hayashi, M., Kaku, M., Katuen, S., Kobayashi, H., and Kawai, F. (2008). Degradation of a terephthalate-containing polyester by thermophilic *Actinomycetes* and *Bacillus* species derived from composts *Journal of Polymers and the Environment* 16**,** 103-108. doi:10.1007/s10924-008-0088-5.

Huang, Y.-C., Chen, G.-H., Chen, Y.-F., Chen, W.-L., and Yang, C.-H. (2010). Heterologous expression of thermostable acetylxylan esterase gene from *Thermobifida fusca* and its synergistic action with xylanase for the production of xylooligosaccharides. *Biochemical and Biophysical Research Communications* 400**,** 718-723. doi:<https://doi.org/10.1016/j.bbrc.2010.08.136>.

Joo, S., Cho, I.J., Seo, H., Son, H.F., Sagong, H.-Y., Shin, T.J., Choi, S.Y., Lee, S.Y., and Kim, K.-J. (2018). Structural insight into molecular mechanism of poly(ethylene terephthalate) degradation. *Nature Communications* 9**,** 382. doi:10.1038/s41467-018-02881-1.

Kawabata, T., Oda, M., and Kawai, F. (2017). Mutational analysis of cutinase-like enzyme, Cut190, based on the 3D docking structure with model compounds of polyethylene terephthalate. *Journal of Bioscience and Bioengineering* 124**,** 28-35. doi:<https://doi.org/10.1016/j.jbiosc.2017.02.007>.

Kawai, F., Kawabata, T., and Oda, M. (2019). Current knowledge on enzymatic PET degradation and its possible application to waste stream management and other fields *Applied Microbiology and Biotechnology* 103**,** 4253-4268. doi:10.1007/s00253-019-09717-y.

Kawai, F., Oda, M., Tamashiro, T., Waku, T., Tanaka, N., Yamamoto, M., Mizushima, H., Miyakawa, T., and Tanokura, M. (2014). A novel Ca2+-activated, thermostabilized polyesterase capable of hydrolyzing polyethylene terephthalate from *Saccharomonospora viridis* AHK190. *Applied Microbiology and Biotechnology* 98**,** 10053-10064. doi:10.1007/s00253-014-5860-y.

Kim, J.W., Park, S.-B., Tran, Q.-G., Cho, D.-H., Choi, D.-Y., Lee, Y.J., and Kim, H.-S. (2020). Functional expression of polyethylene terephthalate-degrading enzyme (PETase) in green microalgae. *Microbial Cell Factories* 19**,** 97. doi:10.1186/s12934-020-01355-8.

Kleeberg, I., Welzel, K., Vandenheuvel, J., Müller, R.J., and Deckwer, W.D. (2005). Characterization of a new extracellular hydrolase from *Thermobifida fusca* degrading aliphatic−aromatic copolyesters *Biomacromolecules* 6**,** 262-270. doi:10.1021/bm049582t.

Krasteva, P.V., Bernal-Bayard, J., Travier, L., Martin, F.A., Kaminski, P.-A., Karimova, G., Fronzes, R., and Ghigo, J.-M. (2017). Insights into the structure and assembly of a bacterial cellulose secretion system. *Nature Communications* 8**,** 2065. doi:10.1038/s41467-017-01523-2.

Liu, B., He, L., Wang, L., Li, T., Li, C., Liu, H., Luo, Y., and Bao, R. (2018). Protein crystallography and site-direct mutagenesis analysis of the Poly(ethylene terephthalate) hydrolase PETase from *Ideonella sakaiensis*. *ChemBioChem* 19**,** 1471-1475. doi:<https://doi.org/10.1002/cbic.201800097>.

Lykidis, A., Mavromatis, K., Ivanova, N., Anderson, I., Land, M., Dibartolo, G., Martinez, M., Lapidus, A., Lucas, S., Copeland, A., Richardson, P., Wilson, D.B., and Kyrpides, N. (2007). Genome sequence and analysis of the soil cellulolytic Actinomycete *Thermobifida fusca* YX *Journal of Bacteriology* 189**,** 2477. doi:10.1128/JB.01899-06.

Ma, Y., Yao, M., Li, B., Ding, M., He, B., Chen, S., Zhou, X., and Yuan, Y. (2018). Enhanced poly(ethylene terephthalate) hydrolase activity by protein engineering. *Engineering* 4**,** 888-893. doi:<https://doi.org/10.1016/j.eng.2018.09.007>.

Meyer-Cifuentes, I.E., Werner, J., Jehmlich, N., Will, S.E., Neumann-Schaal, M., and Öztürk, B. (2020). Synergistic biodegradation of aromatic-aliphatic copolyester plastic by a marine microbial consortium. *Nature Communications* 11**,** 5790. doi:10.1038/s41467-020-19583-2.

Molitor, R., Bollinger, A., Kubicki, S., Loeschcke, A., Jaeger, K.-E., and Thies, S. (2020). Agar plate-based screening methods for the identification of polyester hydrolysis by *Pseudomonas* species. *Microbial Biotechnology* 13**,** 274-284. doi:10.1111/1751-7915.13418.

Moog, D., Schmitt, J., Senger, J., Zarzycki, J., Rexer, K.H., Linne, U., Erb, T., and Maier, U.G. (2019). Using a marine microalga as a chassis for polyethylene terephthalate (PET) degradation. *Microbial Cell Factories* 18**,** 171. doi:10.1186/s12934-019-1220-z.

Müller, R.-J., Schrader, H., Profe, J., Dresler, K., and Deckwer, W.-D. (2005). Enzymatic degradation of poly(ethylene terephthalate): Rapid hydrolyse using a hydrolase from *T. fusca*. *Macromolecular Rapid Communications* 26**,** 1400-1405. doi:10.1002/marc.200500410.

Oda, M., Yamagami, Y., Inaba, S., Oida, T., Yamamoto, M., Kitajima, S., and Kawai, F. (2018). Enzymatic hydrolysis of PET: functional roles of three Ca2+ ions bound to a cutinase-like enzyme, Cut190*, and its engineering for improved activity. *Applied Microbiology and Biotechnology* 102**,** 10067-10077. doi:10.1007/s00253-018-9374-x.

Palm, G.J., Reisky, L., Böttcher, D., Müller, H., Michels, E.a.P., Walczak, M.C., Berndt, L., Weiss, M.S., Bornscheuer, U.T., and Weber, G. (2019). Structure of the plastic-degrading *Ideonella sakaiensis* MHETase bound to a substrate. *Nature Communications* 10**,** 1717. doi:10.1038/s41467-019-09326-3.

Ribitsch, D., Acero, E.H., Greimel, K., Eiteljoerg, I., Trotscha, E., Freddi, G., Schwab, H., and Guebitz, G.M. (2012a). Characterization of a new cutinase from *Thermobifida alba* for PET-surface hydrolysis. *Biocatalysis and Biotransformation* 30**,** 2-9. doi:10.3109/10242422.2012.644435.

Ribitsch, D., Herrero Acero, E., Greimel, K., Dellacher, A., Zitzenbacher, S., Marold, A., Díaz Rodríguez, R., Steinkellner, G., Gruber, K., Schwab, H., and Guebitz, G. (2012b). A new esterase from *Thermobifida halotolerans* hydrolyses Polyethylene Terephthalate (PET) and Polylactic Acid (PLA) *Polymers* 4**,** 617-619. doi:10.3390/polym4010617.

Ribitsch, D., Heumann, S., Trotscha, E., Herrero Acero, E., Greimel, K., Leber, R., Birner-Gruenberger, R., Deller, S., Eiteljoerg, I., Remler, P., Weber, T., Siegert, P., Maurer, K.-H., Donelli, I., Freddi, G., Schwab, H., and Guebitz, G. (2011). Hydrolysis of Polyethyleneterephthalate by p-Nitrobenzylesterase from *Bacillus subtilis*. *Biotechnology progress* 27. doi:10.1002/btpr.610.

Ronkvist, Å.M., Xie, W., Lu, W., and Gross, R.A. (2009). Cutinase-catalyzed hydrolysis of poly(ethylene terephthalate). *Macromolecules* 42**,** 5128-5138. doi:10.1021/ma9005318.

Roth, C., Wei, R., Oeser, T., Then, J., Föllner, C., Zimmermann, W., and Sträter, N. (2014). Structural and functional studies on a thermostable polyethylene terephthalate degrading hydrolase from *Thermobifida fusca* *Applied Microbiology and Biotechnology* 98**,** 7815-7823. doi:10.1007/s00253-014-5672-0.

Sagong, H.-Y., Seo, H., Kim, T., Son, H.F., Joo, S., Lee, S.H., Kim, S., Woo, J.-S., Hwang, S.Y., and Kim, K.-J. (2020). Decomposition of the PET Film by MHETase using Exo-PETase function *ACS Catalysis* 10**,** 4805-4812. doi:10.1021/acscatal.9b05604.

Shirke, A.N., White, C., Englaender, J.A., Zwarycz, A., Butterfoss, G.L., Linhardt, R.J., and Gross, R.A. (2018). Stabilizing leaf and branch compost cutinase (LCC) with glycosylation: Mechanism and effect on PET hydrolysis *Biochemistry* 57**,** 1190-1200. doi:10.1021/acs.biochem.7b01189.

Silva, C., Da, S., Silva, N., Matamá, T., Araújo, R., Martins, M., Chen, S., Chen, J., Wu, J., Casal, M., and Cavaco-Paulo, A. (2011). Engineered *Thermobifida fusca* cutinase with increased activity on polyester substrates. *Biotechnology Journal* 6**,** 1230-1239. doi:<https://doi.org/10.1002/biot.201000391>.

Son, H.F., Cho, I.J., Joo, S., Seo, H., Sagong, H.-Y., Choi, S.Y., Lee, S.Y., and Kim, K.-J. (2019). Rational protein engineering of thermo-stable PETase from *Ideonella sakaiensis* for highly efficient PET degradation *ACS Catalysis* 9**,** 3519-3526. doi:10.1021/acscatal.9b00568.

Su, L., Woodard, R.W., Chen, J., and Wu, J. (2013). Extracellular location of *Thermobifida fusca* cutinase expressed in *Escherichia coli* BL21(DE3) without mediation of a signal peptide *Applied and environmental microbiology* 79**,** 4192-4198. doi:10.1128/AEM.00239-13.

Sulaiman, S., Yamato, S., Kanaya, E., Kim, J.-J., Koga, Y., Takano, K., and Kanaya, S. (2012). Isolation of a novel cutinase homolog with polyethylene terephthalate-degrading activity from leaf-branch compost by using a metagenomic approach. *Applied and environmental microbiology* 78**,** 1556-1562. doi:10.1128/AEM.06725-11.

Then, J., Wei, R., Oeser, T., Barth, M., Belisário-Ferrari, M.R., Schmidt, J., and Zimmermann, W. (2015). Ca2+ and Mg2+ binding site engineering increases the degradation of polyethylene terephthalate films by polyester hydrolases from *Thermobifida fusca*. *Biotechnol J* 10**,** 592-598. doi:10.1002/biot.201400620.

Then, J., Wei, R., Oeser, T., Gerdts, A., Schmidt, J., Barth, M., and Zimmermann, W. (2016). A disulfide bridge in the calcium binding site of a polyester hydrolase increases its thermal stability and activity against polyethylene terephthalate. *FEBS Open Bio* 6**,** 425-432. doi:10.1002/2211-5463.12053.

Thumarat, U., Nakamura, R., Kawabata, T., Suzuki, H., and Kawai, F. (2012). Biochemical and genetic analysis of a cutinase-type polyesterase from a thermophilic *Thermobifida alba* AHK119. *Applied Microbiology and Biotechnology* 95**,** 419-430. doi:10.1007/s00253-011-3781-6.

Tournier, V., Topham, C.M., Gilles, A., David, B., Folgoas, C., Moya-Leclair, E., Kamionka, E., Desrousseaux, M.L., Texier, H., Gavalda, S., Cot, M., Guémard, E., Dalibey, M., Nomme, J., Cioci, G., Barbe, S., Chateau, M., André, I., Duquesne, S., and Marty, A. (2020). An engineered PET depolymerase to break down and recycle plastic bottles. *Nature* 580**,** 216-219. doi:10.1038/s41586-020-2149-4.

Vita, N., Borne, R., and Fierobe, H.-P. (2020). Cell-surface exposure of a hybrid 3-cohesin scaffoldin allowing the functionalization of *Escherichia coli* envelope. *Biotechnology and Bioengineering* 117**,** 626-636. doi:<https://doi.org/10.1002/bit.27242>.

Wei, R., Oeser, T., Schmidt, J., Meier, R., Barth, M., Then, J., and Zimmermann, W. (2016). Engineered bacterial polyester hydrolases efficiently degrade polyethylene terephthalate due to relieved product inhibition. *Biotechnology and Bioengineering* 113**,** 1658-1665. doi:<https://doi.org/10.1002/bit.25941>.

Wei, R., Oeser, T., Then, J., Kühn, N., Barth, M., Schmidt, J., and Zimmermann, W. (2014). Functional characterization and structural modeling of synthetic polyester-degrading hydrolases from *Thermomonospora curvata*. *AMB Express* 4**,** 44-44. doi:10.1186/s13568-014-0044-9.

Xi, X., Ni, K., Hao, H., Shang, Y., Zhao, B., and Qian, Z. (2021). Secretory expression in *Bacillus subtilis* and biochemical characterization of a highly thermostable polyethylene terephthalate hydrolase from bacterium HR29. *Enzyme and Microbial Technology* 143**,** 109715. doi:<https://doi.org/10.1016/j.enzmictec.2020.109715>.

Yan, F., Wei, R., Cui, Q., Bornscheuer, U.T., and Liu, Y.-J. (2020). Thermophilic whole-cell degradation of polyethylene terephthalate using engineered *Clostridium thermocellum*. *Microbial Biotechnology* n/a. doi:<https://doi.org/10.1111/1751-7915.13580>.

Yoshida, S., Hiraga, K., Takehana, T., Taniguchi, I., Yamaji, H., Maeda, Y., Toyohara, K., Miyamoto, K., Kimura, Y., and Oda, K. (2016). A bacterium that degrades and assimilates poly(ethylene terephthalate). *Science* 351**,** 1196. doi:10.1126/science.aad6359.
